# High-mobility group box 1 and the receptor for advanced glycation end products contribute to lung injury during *Staphylococcus aureus* pneumonia

**DOI:** 10.1186/cc13162

**Published:** 2013-12-16

**Authors:** Ahmed Achouiti, Anne Jan van der Meer, Sandrine Florquin, Huan Yang, Kevin J Tracey, Cornelis van ’t Veer, Alex F de Vos, Tom van der Poll

**Affiliations:** 1 Center for Experimental and Molecular Medicine, Meibergdreef 9, Room G2-130, Amsterdam 1105 AZ, The Netherlands; 2 Center for Infection and Immunity, Academic Medical Center, University of Amsterdam, Meibergdreef 9, Room G2-130, Amsterdam 1105 AZ, The Netherlands; 3 Department of Pathology; Academic Medical Center, University of Amsterdam, Amsterdam, The Netherlands; 4 Center for Biomedical Science, The Feinstein Institute for Medical Research, Manhasset, New York, USA; 5 Division of Infectious Diseases, Academic Medical Center, University of Amsterdam, Amsterdam, The Netherlands

## Abstract

**Introduction:**

*Staphylococcus (S.) aureus* has emerged as an important cause of necrotizing pneumonia. Lung injury during *S. aureus* pneumonia may be enhanced by local release of damage associated molecular patterns such as high-mobility group box 1 (HMGB1). In the current study we sought to determine the functional role of HMGB1 and its receptors, toll-like receptor 4 (TLR4) and the receptor for advanced glycation end products (RAGE), in the injurious host response to *S. aureus* pneumonia.

**Methods:**

Pneumonia was induced in wild type (Wt), TLR4 deficient (*tlr4*^*−/−*^) and RAGE deficient (*rage*^*−/−*^) mice by intranasal inoculation of 1 × 10^7^ colony-forming units (CFU) of a USA300 *S. aureus*. In a separate set of experiments, Wt mice were injected intraperitoneally with a monoclonal anti-HMGB1 antibody or an isotype matched control antibody immediately before and every 24 hours after intranasal infection of *S. aureus.* Mice were sacrificed at 6, 24, 48 or 72 hours after infection for harvesting of blood and organs.

**Results:**

*S. aureus* pneumonia was associated with HMGB1 release in the bronchoalveolar compartment peaking after 24 hours. Anti-HMGB1 attenuated lung pathology and protein leak and reduced interleukin-1β release 6 hours after infection, but not at later time points. RAGE deficiency more modestly attenuated lung pathology without influencing protein leak, while TLR4 deficiency did not impact on lung injury.

**Conclusion:**

These data suggest that HMGB1 and RAGE, but not TLR4, contribute to lung injury accompanying the early phase of *S. aureus* pneumonia*.*

## Introduction

The Gram-positive bacterium *Staphylococcus (S.) aureus* is a frequent colonizer of the human body, and when the opportunity arises, is able to cause a wide array of clinical syndromes [1]. Infections caused by this pathogen impose a high burden on healthcare, largely due to the increasing incidence of antibiotic resistance [2]. Over the past few years, highly virulent methicillin-resistant *S. aureus* strains, in particular USA300, have become prevalent in the community as well [2] and have emerged as an important cause of (necrotizing) pneumonia [3]. Pneumonia caused by these strains have a fulminant onset determined by staphylococcal virulence factors and the nature of the immune response [3,4]. More insight into pathogenic mechanisms that influence the outcome of lower airway infection by *S. aureus* could help in the development of new (immunomodulating) therapies.

Staphylococcal pneumonia is associated with a massive influx of neutrophils and release of cytotoxic granular proteins [5-7]. Together with invasive infection, intense host defense mechanisms likely contribute to lung tissue damage and release of damage-associated molecular patterns (DAMPs) [4,7,8]. Pattern-recognition receptors that engage with these self-derived proteins may contribute to the severity of pneumonia as they perpetuate (excessive) inflammation. High-mobility group box 1 (HMGB1) is a DAMP that may be of particular interest as it is associated with delayed and sustained release during infection [9]. HMGB1 is a highly conserved non-histone nuclear protein, which is either released passively during cell injury or secreted actively upon inflammatory stimuli [9]. Depending on specific posttranslational redox modifications HMGB1 can act as a cytokine via receptors such as the receptor for advanced glycation end products (RAGE) and toll-like receptor (TLR)4 or as a chemotactic factor by forming a heterocomplex with the chemokine CXCL12 via the chemokine receptor CXCR4 [10].

In this study we investigated the role of HMGB1 in experimentally induced *S. aureus* pneumonia. This newly developed mouse model of pneumonia is associated with severe pulmonary inflammation and massive influx of neutrophils. In order to study the role of HMGB1 in the pathogenesis of *S. aureus* lung infection we inoculated wild-type (Wt) mice with a USA300 strain of *S. aureus* and treated animals with a control or an anti-HMGB1 antibody. In addition, we investigated Wt mice and mice deficient for TLR4 or RAGE, the receptors implicated in mediating the proinflammatory effects of HMGB1, after induction of *S. aureus* pneumonia.

## Methods

### Ethics statement

Experiments were carried out in accordance with the Dutch Experiment on Animals Act and approved by the Animal Care and Use Committee of the University of Amsterdam (Permit number: DIX100121).

### Mice

C57Bl/6 Wt mice were purchased from Charles River Laboratories Inc. (Maastricht, the Netherlands). RAGE-deficient (*rage*^*−/−*^) [11] and *tlr4*^*−/−*^ mice [12], backcrossed >10 times to a C57BL/6 background were generated as described and bred in the animal facility of the Academic Medical Center (Amsterdam, the Netherlands).

### Design

Wt, *Rage*^*−/−*^ and *tlr4*^*−/−*^ mice were lightly anesthesized by inhalation of isoflurane (Abbot Laboratories, Queensborough, Kent, UK) and intranasally inoculated with a sub-lethal dose of 1 × 10^7^ *S. aureus* USA300 (BK 11540) in a 5-μl saline solution (n = 7 to 8 per strain). This sub-lethal dose was determined in a pilot study: mice that were intranasally inoculated with 1 × 10^8^ *S. aureus* died after 24 hours, whereas mice that were infected with 1 × 10^7^ *S. aureus* were clinically ill, but remained alive until 72 hours after infection (data not shown). In separate experiments Wt mice were injected intraperitoneally with either 150 μg monoclonal anti-HMGB1 (2G7, IgG2b; Cornerstone Therapeutics, Cary, NC, USA) or an isotype-matched control antibody (MOPC-195, IgG2b; Sigma, St. Louis, MO, USA) in 100 μl PBS) immediately before and every 24 hours after intranasal infection of *S. aureus.* The dose of this anti-HMGB1 monoclonal antibody was selected according to a previous study and is able to effectively inhibit HMGB1 induced pathology in a mouse model of infectious lung injury [13]. Mice were sacrificed at 6, 24, 48 or 72 hours after infection.

### Bacterial cultures

Measurements of bacterial loads were done as described previously [14]. In brief, blood was drawn into heparinized tubes, bronchoalveolar lavage (BAL) fluid was obtained as described below and organs were removed aseptically and homogenised in four volumes of sterile isotonic saline using a tissue homogenizer (Biospec Products, Bartlesville, OK, USA). To determine bacterial loads, ten-fold dilutions were plated on blood agar plates and incubated at 37°C for 16 hours.

### Bronchoalveolar lavage

The trachea and bronchi were exposed through a midline incision. The left main bronchus was ligated and the trachea was cannulated with a sterile 22-gauge Abbocath-T catheter (Abbott Laboratories, Sligo, Ireland). Unilateral, right-sided BAL was performed by instilling three 0.3-ml aliquots of sterile PBS: 0.7 to 0.9 ml of BAL fluid was retrieved per mouse. Total cell numbers in BAL were counted using a Z2 Coulter particle count and size analyzer (Beckman-Coulter, Inc., Miami, FL, USA). BAL fluid differential cell-counts were determined on cytospin preparations stained with a modified Giemsa stain (Diff-Quick; Baxter, McGraw Park, IL, USA).

### Assays

Cytokines and chemokines TNF-α, IL-1β, IL-6, IL-10, keratinocyte-derived chemokine (KC) and macrophage inflammatory protein (MIP)-2 (all R&D systems, Minneapolis, MN, USA) were measured in BAL fluid using specific ELISAs according to manufacturer’s recommendations. Total protein concentrations were measured using a DC protein assay (Bio-Rad Laboratories, Veenendaal, The Netherlands).

### HMGB1 western blot

For western blotting of HMGB1, non-reduced BAL fluid samples from Wt mice were diluted with 3× Laemmli buffer (n = 5 per time point). After heating, samples were run on 15% polyacrylamide SDS gels and subsequently transferred to blotting membrane (polyvinylidene difluoride membranes; Immobilon P, Pharmacia, Piscataway, NJ, USA). Following blocking with 5% nonfat dry milk proteins (Protifar; Nutricia, Zoetermeer, The Netherlands) in 0.1% Tween 20 PBS (PBS-T), membranes were washed and incubated overnight in 1 μg/ml primary rabbit anti-HMGB1 polyclonal antibody (Abcam, Cambridge, UK) in 1% nonfat dry milk proteins in PBS-T at 4°C. After washing with PBS-T, membranes were probed with anti-rabbit-HRP-conjugated secondary antibody (Cell Signaling Technology, Danvers, MA, USA) for 1 hour at room temperature in 1% bovine serum albumin in PBS-T. After washing with PBS-T, membranes were incubated with Lumi-Light Plus Western Blotting Substrate (Roche, Mijdrecht, The Netherlands) and positive bands were detected using an LAS3000 Luminescent image Analyzer dark box (Fujifilm, Tokyo, Japan). Band densities were determined using AIDA Image analyzer software (Raytest, Straubenhardt, Germany) and expressed as a percentage of band densities of naive control samples.

### Histology

Lung pathology scores were determined as described before [14]. In brief, the left lung lobe was harvested at the indicated time points, fixed in 10% buffered formalin, and embedded in paraffin: 4-μm sections were then stained with H&E and analyzed by a pathologist blinded for groups. To score lung inflammation and damage, the entire lung surface was analyzed with respect to the following parameters: bronchitis, edema, interstitial inflammation, intra-alveolar inflammation, pleuritis, endothelialitis and percentage of the lung surface demonstrating confluent inflammatory infiltrate. Each parameter was graded 0 to 4, with 0 representing absent and 4 representing severe. The total pathology score was expressed as the sum of the score for all parameters.

### Statistical analysis

Data are expressed as box-and-whisker diagrams depicting the smallest observation, lower quartile, median, upper quartile and largest observation unless indicated otherwise. Differences between groups of mice were analyzed by the Mann-Whitney *U*-test. Analyses were done using GraphPad Prism version 5.0, Graphpad Software (San Diego, CA, USA). Values of *P* <0.05 were considered significantly different.

## Results

### HMGB1 is released in bronchoalveolar lavage fluid during staphylococcal pneumonia

**Figure 1:**
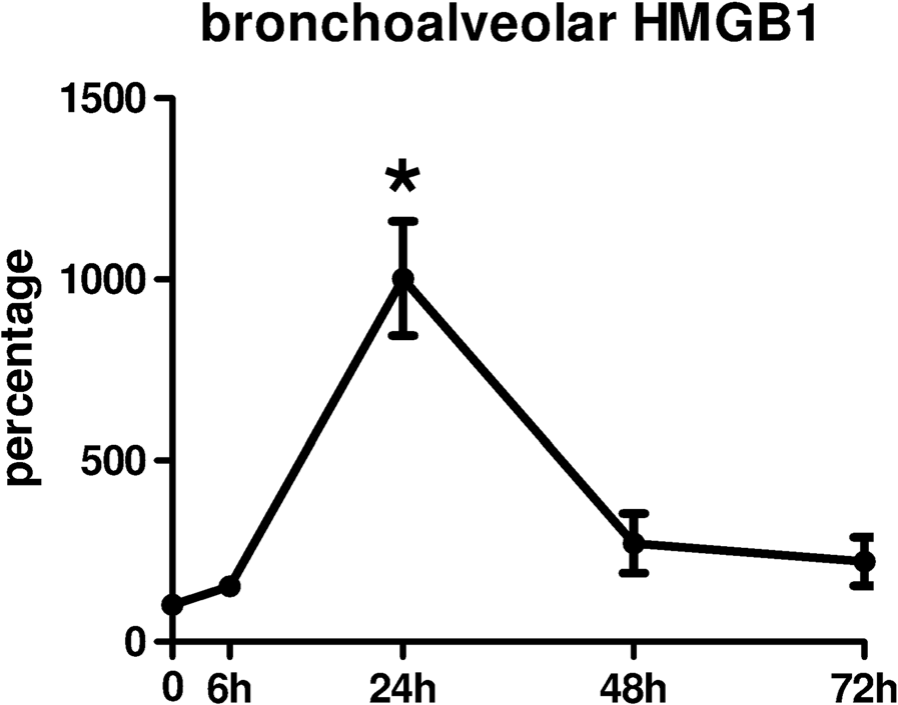
**High mobility group box 1 (HMGB1) concentrations in mouse bronchoalveolar lavage (BAL) fluid during *S. aureus *pneumonia.** Wt mice were intranasally infected with 1 × 10^7^ colony forming units (CFU) *S. aureus* and euthanized after 6, 24, 48 and 72 hours. HMGB1 concentrations in BAL fluid were quantified by densometric analysis of HMGB1 western blots and expressed as a percentage of the mean density of control BAL fluid samples (0 hours). Data represent the means ± standard error of the mean (n = 5 mice per time point). ^*^P <0.05 versus naïve mice (0 hours).

To examine HMGB1 release in murine *S. aureus* pneumonia we performed a western blot by running non-reduced BAL fluid samples harvested at various time points in Wt mice after intranasal inoculation of a sublethal dose of *S. aureus.* Uninfected mice displayed almost no detectable levels of HMGB1. HMGB1 bands were detected as early as 6 hours post infection. HMGB1 concentrations peaked after 24 hours and decreased thereafter (Figure 1).

### HMGB1 and RAGE, but not TLR4, contribute to lung pathology

**Figure 2:**
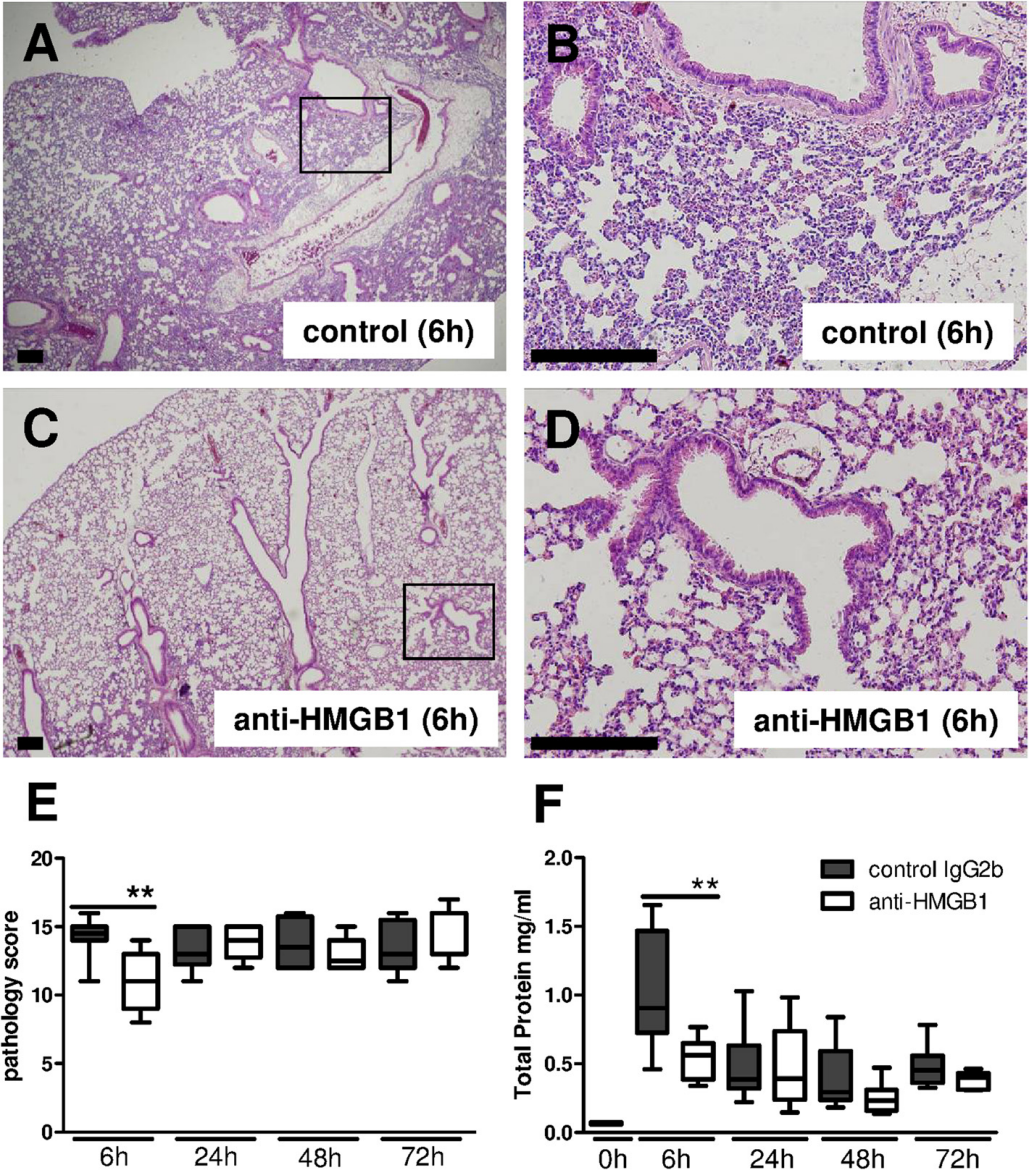
**Anti-high mobility group box 1 (HMGB1)-treated mice show reduced lung pathology early after induction of *S. aureus *pneumonia.** Representative slides of lung H&E staining of control treated **(A)** and anti-HMGB1 treated mice **(C)**, original magnification × 2. The boxed areas are also shown at a higher magnification for controls **(B)** and anti-HMGB1-treated mice **(D)**, original magnification × 10. Scale bars indicate 200 μm. Total pathology scores were determined at the indicated time points post infection in control treated (gray) and anti-HMGB1 treated mice (white) according to the scoring system described in the Methods section **(E)**. Total protein was measured in BAL fluid from control (grey) and anti-HMGB1 treated (white) mice **(F)**. Data are expressed as box-and-whisker diagrams depicting the smallest observation, lower quartile, median, upper quartile and largest observation (7 to 8 mice per group at each time point). ^**^P <0.01 versus control treated mice at the same time point.

**Figure 3:**
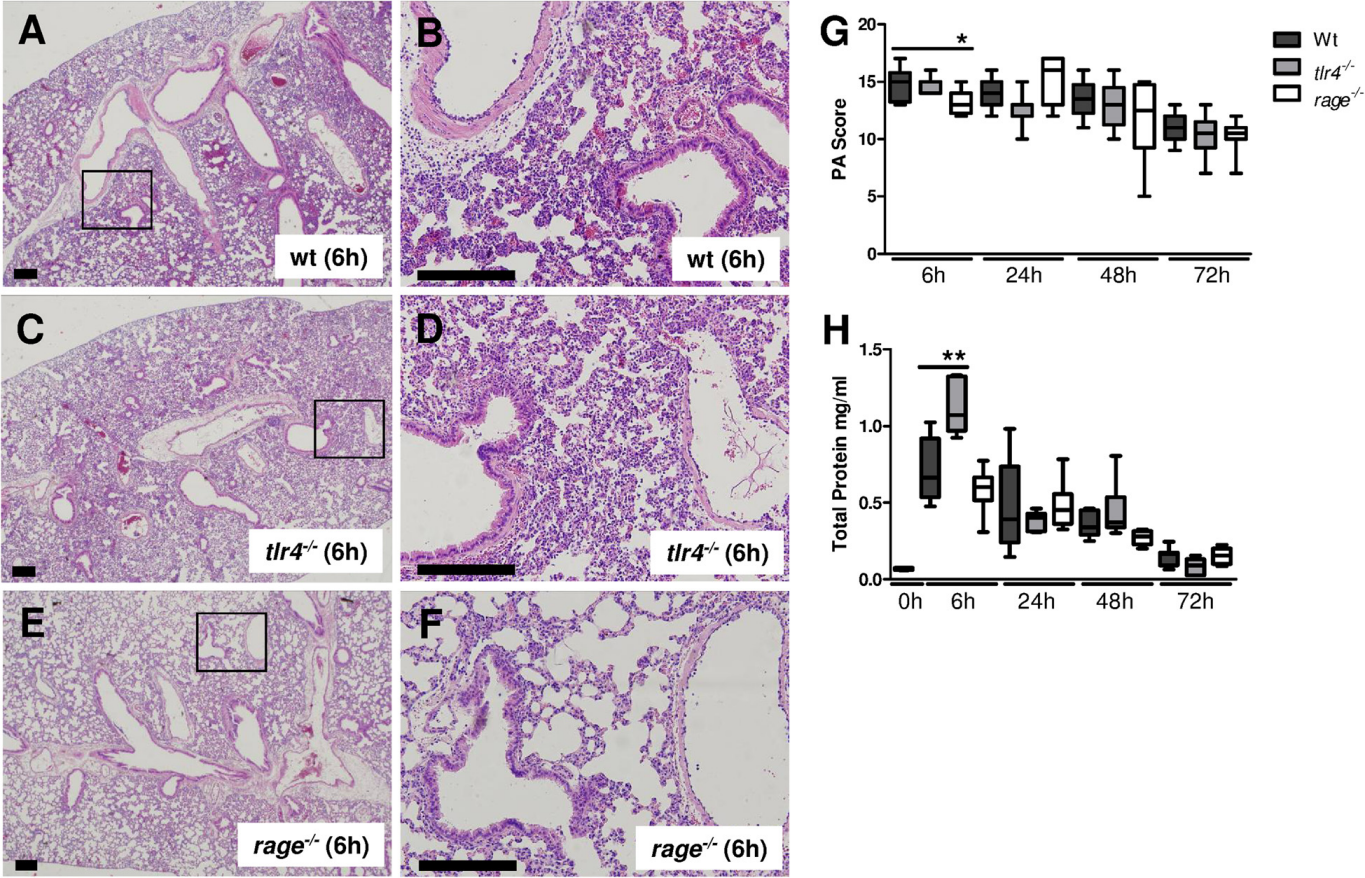
***Receptor for advanced glycation end products *(*Rage*) **^***−/−***^**but not *Toll-like receptor (tlr*)*4***^***−/−***^**mice show reduced lung pathology during *S. aureus *pneumonia.** Representative slides of lung HE staining of wild-type (Wt) **(A)**, *tlr4*^*−/−*^**(C)** and *rage*^*−/−*^ mice **(E)**, original magnification × 2. The boxed areas are also shown at a higher magnification for Wt **(B)***tlr4*^*−/−*^**(D)** and *rage*^*−/−*^ mice **(F)**, original magnification × 10. Scale bars indicate 200 μm. Total pathology scores at indicated time points after intranasal infection with *S. aureus* in Wt (dark gray), *tlr4*^*−/−*^ (light gray) and *rage*^*−/−*^ mice (white) were determined according to the scoring system described in the Methods section **(G)**. Total protein was measured in bronchoalveolar lavage (BAL) fluid from Wt (dark gray), *tlr4*^*−/−*^ (light gray) and *rage*^*−/−*^ mice (white) at the indicated time points after intranasal *S. aureus* infection **(H)**. Data are expressed as box-and-whisker diagrams depicting the smallest observation, lower quartile, median, upper quartile and largest observation (7 to 8 mice per group at each time point). ^*^P <0.05, ^**^P <0.01 versus Wt mice at the same time point.

To evaluate the role of HMGB1, RAGE and TLR4 in the severity of lung pathology, we analyzed pulmonary inflammation and injury in lung-tissue slides obtained from anti-HMGB1 treated, *rage*^*−/−*^ and *tlr4*^*−/−*^ mice and their respective control animals at 6, 24, 48 and 72 hours after infection with *S. aureus* (Figures 2 and 3). Total pathology scores were quantified according to the scoring system described in the Methods section. Mice already displayed signs of acute lung injury including interstitial inflammation, endothelialitis, bronchitis and pleuritis after 6 hours; the extent of lung pathology upon histological examination remained relatively stable thereafter. At 6 hours post infection, anti-HMGB1 treatment reduced total pathology scores (*P* <0.01 versus Wt mice), which was mostly caused by a reduction of lung edema compared to control antibody treatment (Figure 2A-E). In accordance, anti-HMGB1 attenuated protein leak into the bronchoalveolar space at this early time point, as reflected by lower total protein concentrations in BAL fluid (*P* <0.01 versus Wt mice). At later time points anti-HMGB1 did not impact on the extent of lung pathology or protein leak.

TLR4 deficiency did not influence lung pathology (Figure 3). *Rage*^*−/−*^ mice, however, displayed modestly attenuated lung pathology at 6 hours post infection (*P* <0.05 versus Wt mice), which was primarily caused by reduced pleuritis. Although total lung pathology scores were similar in *rage*^*−/−*^ and Wt mice at later time points, the former mouse strain did show significantly smaller areas of confluent inflammatory infiltrates at 48 hours after infection (17 ± 5% of their lung surface versus 31 ± 2% in Wt mice, mean ± standard error of the mean, *P* <0.05, Additional file 1: Figure S1). Remarkably, while RAGE deficiency did not impact on total protein levels in BAL fluid, *tlr4*^*−/−*^ mice displayed an increase of BAL fluid total protein content 6 hours after infection (*P* <0.01 versus Wt mice). In conclusion, these data indicate that RAGE and HMGB1 contribute to the extent of lung pathology in *S. aureus* pneumonia, but, considering their differential impact on histological features, most likely via different mechanisms.

### HMGB1 or RAGE do not influence neutrophil influx during staphylococcal pneumonia

One of the hallmarks in *S. aureus* pneumonia is a massive influx of neutrophils into the lung parenchyma [5,6]. Anti-HMGB1 did not influence neutrophil counts in BAL fluid at any time after infection with *S. aureus* (Table 1). Similarly, RAGE or TLR4 deficiency did not impact on neutrophil influx, except for modestly lower neutrophil numbers in BAL fluid of *tlr4*^*−/−*^ mice harvested 72 hours after infection (Table 2, *P* <0.05 versus Wt mice).

**Table 1:** Influx of neutrophils in bronchiolar lavage fluid of control or anti-HMGB1-treated mice

**Time point (t)**	**Control IgG2b**	**Anti-HMGB1**
t = 6 h	508 (390 to 1169)	520 (207 to 736)
t = 24 h	434 (391 to 580)	665 (450 to 934)
t = 48 h	1376(1054 to 1796)	1339 (1147 to 1770)
t = 72 h	600 (516 to 891)	519(425 to 841)

Neutrophil numbers (×1,000/ml) in BAL fluid at 6, 24, 48 and 72 hours after intranasal *S. aureus* infection in control and anti-HMGB1-treated mice. Data are medians (interquartile ranges); n = 7 to 8 mice per group per time point. HMGB1, High mobility group box 1.

**Table 2:** **Influx of neutrophils in bronchiolar lavage fluid of wild-type, *tlr4***^***−/− ***^**or *Rage***^***−/− ***^**mice**

**Time point (t)**	**Wild-type**	***Tlr4***^***−/−***^	***Rage***^***−/−***^
t = 6 h	239 (217 to 310)	397 (221 to 535)	526 (350 to 647)
t = 24 h	298 (250 to 380)	236 (196 to 275)	342 (262 to 404)
t = 48 h	429 (218 to 652)	522 (421 to 756)	395 (314 to 541)
t = 72 h	146 (99 to 190)	90 (81 to 132)	76 (47 to 104)^*^

Neutrophil numbers (×1000/ml) in bronchiolar lavage fluid at 6, 24, 48 and 72 hours after intranasal *S. aureus* infection in wild-type (Wt), *Toll-like receptor* (*tlr*)*4*^*−/−*^ and *Receptor for advanced glycation end products* (*Rage)*^*−/−*^ mice. Data are medians (interquartile ranges); n = 7 to 8 mice per group per time point. ^*^P <0.05 versus Wt mice at the same time point.

### RAGE and HMGB1, but not TLR4, contribute to pulmonary cytokine and chemokine release early after infection with *S. aureus*

**Figure 4:**
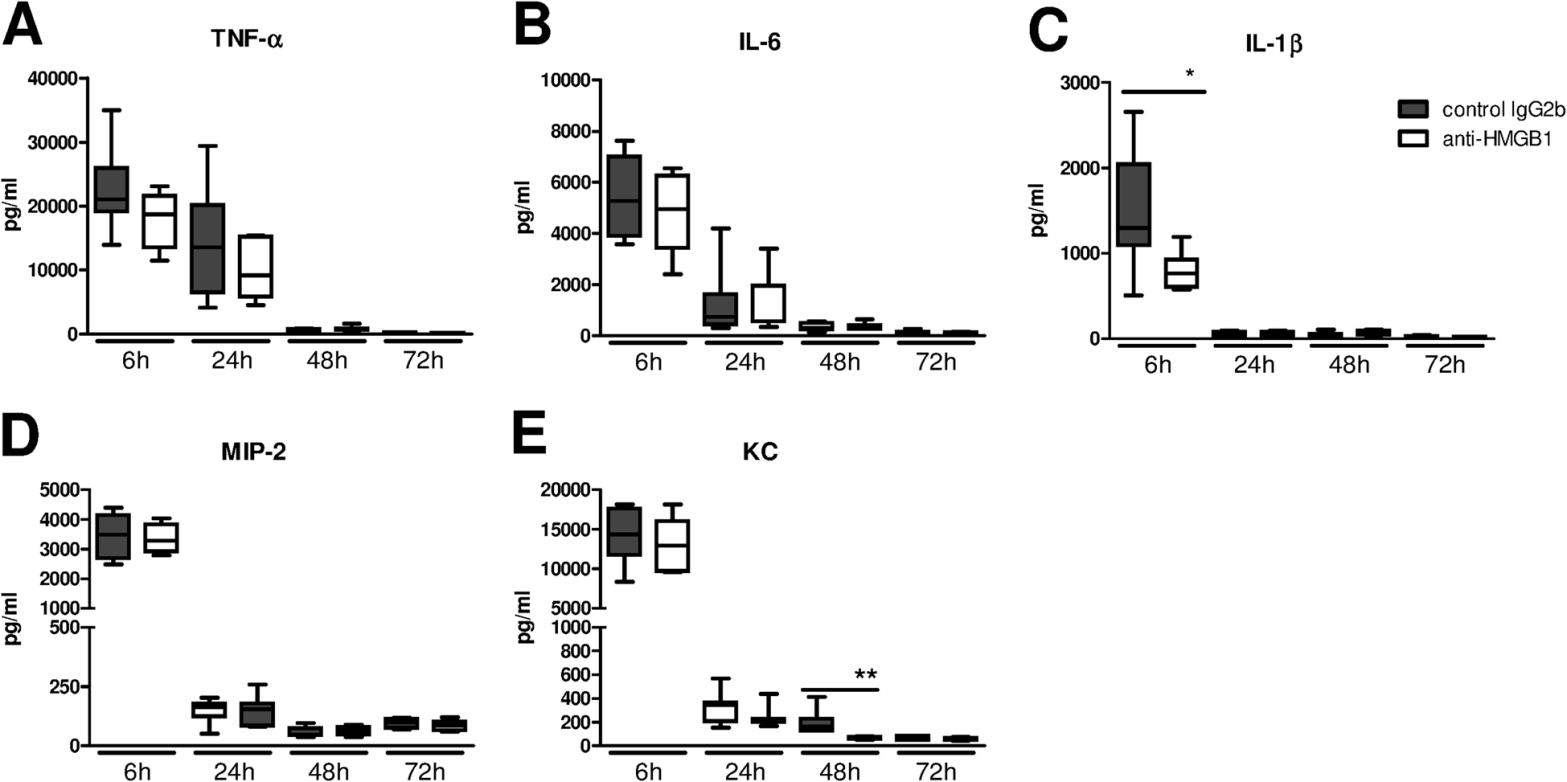
**Anti**-**high mobility group box 1 (HMGB1) treatment reduces IL-1β and keratinocyte**-**derived chemokine (KC) levels in bronchiolar lavage (BAL) fluid after intranasal infection with *S. aureus.*** Cytokine (TNF-α, IL-6 and IL-1β) **(A**-**C)** and chemokine (KC and macrophage inflammatory protein (MIP)-2) **(D**-**E)** levels in BAL fluid at different time points after intranasal infection of 1 × 10^7^ colony-forming units (CFU) *S. aureus* in mice treated with control (gray) and anti-HMGB1 antibodies (white). Data are expressed as box-and-whisker diagrams depicting the median, the smallest observation, lower quartile, median, upper quartile and largest observation (7 to 8 mice per group at each time point). ^*^P <0.05, ^**^P <0.01 versus Wt mice at the same time point.

**Figure 5:**
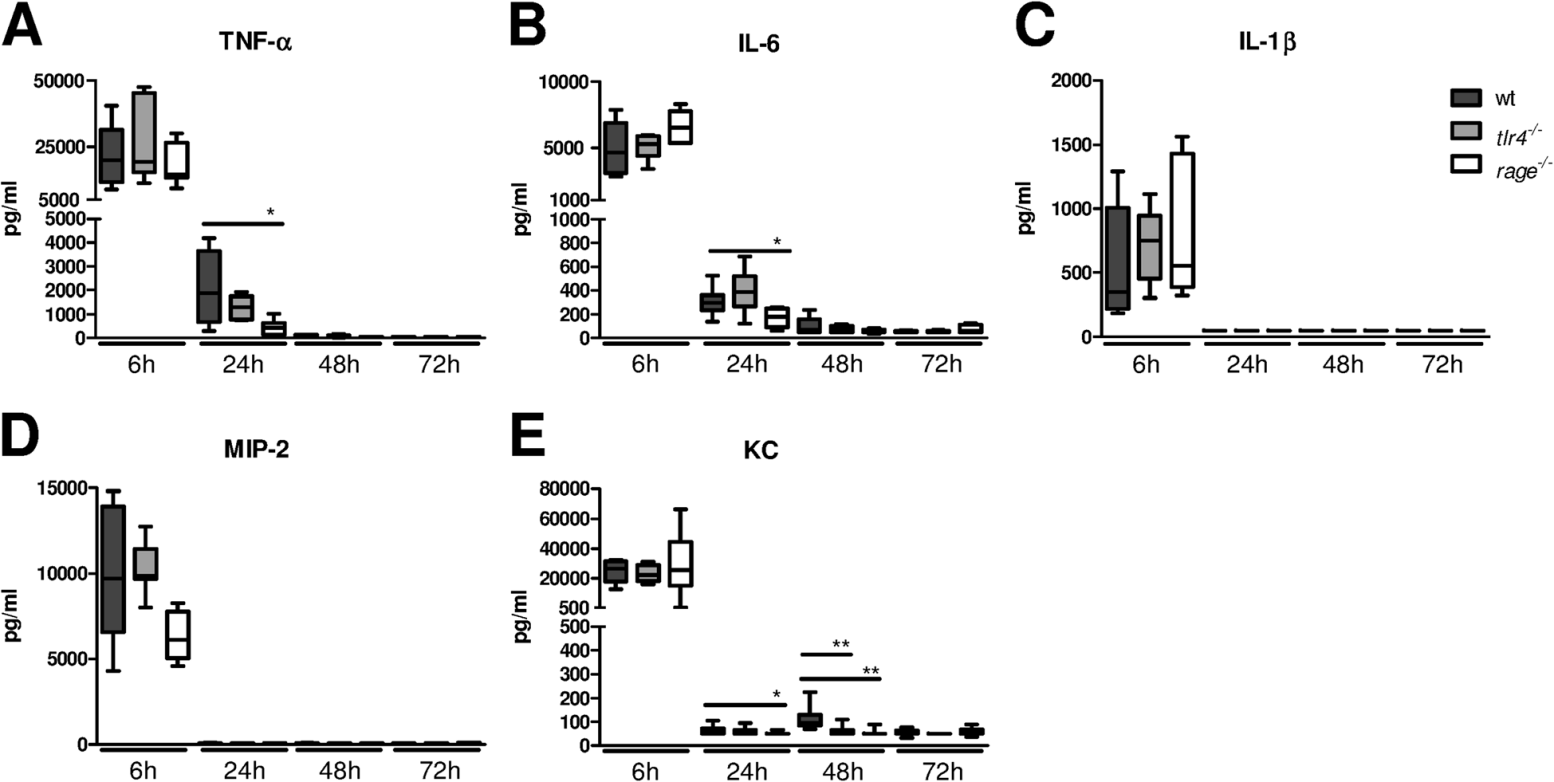
***Receptor for advanced glycation end products *(*Rage*)**^***−/−***^**mice show lower levels of TNF-α and IL-6 in bronchiolar lavage (BAL) fluid during *S. aureus *pneumonia.** Cytokine (TNF-α, IL-6 and IL-1β) **(A**-**C)** and chemokine (keratinocyte-derived chemokine (KC) and macrophage inflammatory protein (MIP)-2) **(D**-**E)** levels 6, 24, 48 and 72 hours after intranasal *S. aureus* infection in Wt (dark gray) and *tlr4*^*−/−*^ (light gray) and *rage*^*−/−*^ mice (white). Data are expressed as box-and-whisker diagrams depicting the smallest observation, lower quartile, median, upper quartile and largest observation (7 to 8 mice per group at each time point). ^*^P <0.05, ^**^P <0.01 versus Wt mice.

To further investigate the role of HMGB1 in lung inflammation during *S. aureus* pneumonia we measured cytokines (TNF-α, IL-1β and IL-6) and chemokines (KC and MIP-2) in BAL fluid from mice treated with anti-HMGB1 or control antibody (Figure 4). All cytokines and chemokines reached peak concentrations at 6 hours after infection. At this time point anti-HMGB1 treatment decreased IL-1β concentrations in BAL fluid (*P* <0.05 versus mice treated with control antibody), without influencing the levels of other proinflammatory mediators. At later time points, cytokine and chemokine levels were low in all mice and not different between groups with the exception of lower KC levels in anti-HMGB1 treated mice at 48 hours. Cytokine levels in BAL fluid of *tlr4*^*−/−*^ mice were similar to those in Wt mice at all time points; *tlr4*^*−/−*^ mice showed lower KC concentrations at 48 hours relative to Wt mice (Figure 5). *Rage*^*−/−*^ mice had lower TNF-α and IL-6 levels at 24 hours of infection and lower KC concentrations at 24 and 48 hours when compared with Wt mice.

### HMGB1 does not influence bacterial clearance during staphylococcal pneumonia

**Figure 6:**
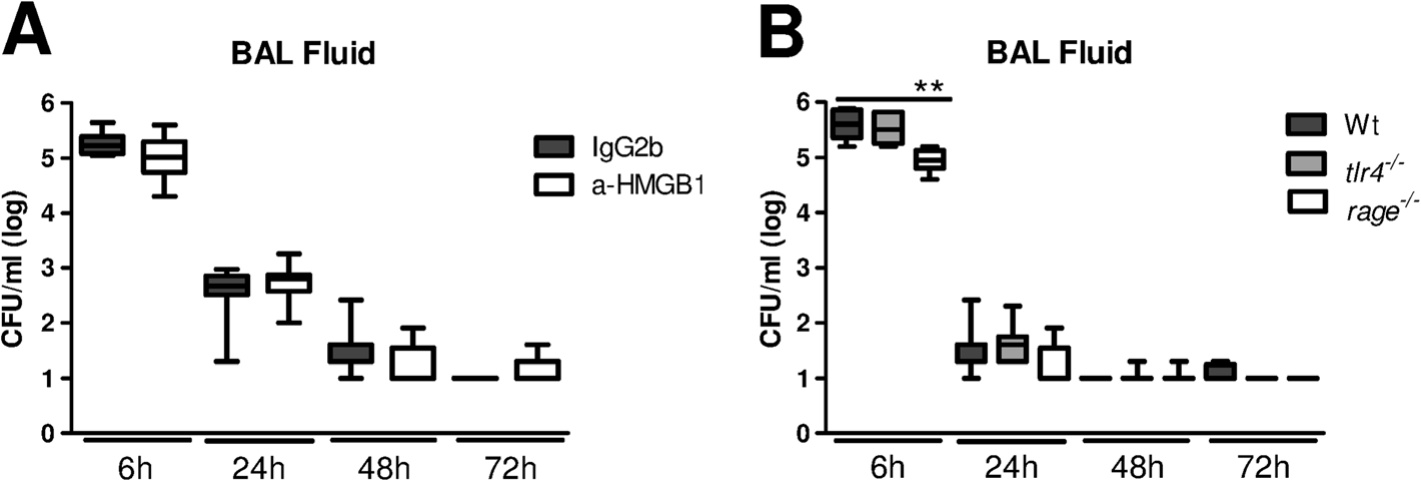
**Bacterial outgrowth in *Receptor for advanced glycation end products *(*Rage*)**^***−/−***^**mice is reduced early after intranasal infection with *S. aureus*.** Bacterial loads after intranasal infection with 1 × 10^7^ colony-forming units (CFU) *S. aureus* in bronchiolar lavage (BAL) fluid **(A)** of mice treated with control (gray) and anti-HMGB1 antibodies (white) and in BAL fluid **(B)** of Wt (dark gray), Toll-like receptor (*tlr*)*4*^*−/−*^ (light gray) and *rage*^*−/−*^ mice (white) at 6, 24, 48 and 72 hours after infection. Data are expressed as box-and-whisker diagrams depicting the median, the smallest observation, lower quartile, median, upper quartile and largest observation (n = 7 to 8 mice per group at each time point). ^**^P <0.01 versus Wt mice at the same time point.

HMGB1 has been shown to impair bacterial clearance during pneumonia caused by *Pseudomonas (P.) aeruginosa*[13,15]. To investigate the role of HMGB1 in clearance of *S. aureus*, we quantified bacterial loads in BAL fluid, liver and blood after intranasal inoculation with 10^7^ *S. aureus* of mice treated with anti-HMGB1 or control antibody. Although HMGB1 was highly present at the primary site of infection during *S. aureus* pneumonia, anti-HMGB1 treatment did not influence bacterial loads at any time point (Figure 6A).

We next investigated the role of TLR4 and RAGE in bacterial clearance. In analogy to the results obtained after anti-HMGB1 treatment, *tlr4*^*−/−*^ mice showed similar bacterial loads as Wt mice at all time points (Figure 6B). *Rage*^*−/−*^ mice displayed 10 times lower bacteria in BAL fluid after 6 hours (*P* <0.01 versus Wt mice), but not at later time points (Figure 6B). Bacteria that disseminated into the circulation were almost immediately cleared in all mice. No differences in bacterial loads in blood or livers were observed between experimental groups (data not shown). Together these data indicate that RAGE impairs clearance of *S. aureus* in the bronchoalveolar compartment early after infection and that HMGB1 has no role herein. Moreover, TLR4 does not contribute antibacterial defense during *S. aureus* pneumonia.

## Discussion

*S. aureus* is a major bacterial pathogen responsible for both healthcare and community-associated infections [1]. Pulmonary infection by *S. aureus* may develop into necrotizing pneumonia and can be very severe as a consequence of both virulence factors and an intense immune response [4]. Lung injury may be further aggravated due to enhancement of inflammation caused by the release of DAMPs such as HMGB1 [4,7,8]. In the current study we intranasally challenged mice with staphylococci to determine the functional role of HMGB1 and two of its proinflammatory receptors in *S. aureus* pneumonia [9]. We showed that *S. aureus* pneumonia is associated with HMGB1 release in the bronchoalveolar compartment peaking after 24 hours. Although relatively little HMGB1 was released at 6 hours, anti-HMGB1 treatment was able to attenuate lung pathology and protein leak, accompanied by lower IL-1β concentrations in BAL fluid at this time point. TLR4, which has been identified as the dominant proinflammatory receptor for HMGB1 [10], had no impact, or very limited impact on the injurious host response during *S. aureus* pneumonia. RAGE deficiency, however, was associated with reduced lung pathology although *rage*^*−/−*^ mice did not phenocopy anti-HMGB1 treated mice, suggesting that different mechanisms are involved. Our results suggest a harmful role for both HMGB1 and RAGE in the development of lung injury during the early phase of severe pneumonia caused by a clinical relevant Gram-positive pathogen.

Previous studies investigated the role of HMGB1 in lung injury associated with Gram-negative pneumonia in mice with various comorbid conditions [13,15]. Anti-HMGB1 treatment attenuated pulmonary neutrophil recruitment and lung injury upon airway infection with *P. aeruginosa* of mice deficient for the cystic fibrosis transmembrane conductance regulator [13] and mice exposed to hyperoxia [15]. In accordance, anti-HMGB1 decreased neutrophil accumulation and lung edema in mice treated with intratracheal endotoxin [16], the proinflammatory component of the Gram-negative bacterial cell wall. Moreover, HMGB1 has been reported to contribute to hemorrhage- and ventilator-induced lung injury in mice [17,18]. To the best of our knowledge, our investigation is the first to study the role of HMGB1 in lung injury elicited by a Gram-positive pathogen. For this we administered a monoclonal anti-HMGB1 antibody previously shown to inhibit HMGB1-induced acute and chronic inflammation in mice [13,19], including pneumonia [13]. Although HMGB1 levels only tended to increase 6 hours after induction of *S. aureus* pneumonia, anti-HMGB1 reduced protein leak and lung edema at this time point, which is in accordance with results obtained during Gram-negative pneumonia [13,15,16], In contrast, we did not find a role for HMGB1 in neutrophil recruitment, which at least in part may be explained by differential pattern recognition receptors and integrins involved in attraction of neutrophils to the lungs by Gram-negative and Gram-positive stimuli [6].

The current model of *S. aureus* pneumonia is associated with a strong early and transient cytokine and chemokine response in the bronchoalveolar space, peaking after 6 hours. Although HMGB1 was not statistically significantly increased at 6 hours, antagonizing this relatively low level of HMGB1 diminished the greatly enhanced IL-1β levels in BAL fluid, which may have contributed to reduced protein leak and pathology at this time point. In accordance, anti-HMGB1 was previously shown to inhibit IL-1β release after airway endotoxin exposure and in pulmonary injury induced by hemorrhage [16,17]. Interestingly enough, we could not find differences in other cytokines at this time point. These seemingly discrepant results are not easily explained and require further investigation. As cytokine production was considerably reduced at time points later than 6 hours in all groups, blockade of HMGB1 from 24 hours onward had only little effect. As such, the brisk induction of inflammatory mediators after intrapulmonary delivery of *S. aureus* is most likely initiated via a TLR2-MyD88 dependent mechanism [20], while the subsequent release of HMGB1 apparently does not sustain this response, although anti-HMGB1 reduced the already low KC levels in BAL fluid at 48 hours.

Several receptors have been implicated in mediating cellular effects of HMGB1 [9]. Purified HMGB1 binds specifically to TLR4 to induce proinflammatory cytokine release by an interaction that requires a cysteine in position 106 [10]. In addition, HMGB1 can mediate proinflammatory effects by binding of partner molecules such as bacterial ligands, extracellular cell free DNA, nucleosomes and IL-1β, through which other pattern recognition receptors can be activated [9]. Our group previously showed that intraperitoneal injection of recombinant HMGB1 induces inflammation by mechanisms that partially depend on TLR4 and RAGE [21]. We showed that TLR4 is not involved in the lung pathology induced by *S. aureus*. We did not expect a direct role for TLR4 in the initiation of lung inflammation induced by *S. aureus*, considering that this pathogen does not express known TLR4 ligands [20]. Of note, however, TLR4 not only can function as a receptor for HMGB1, but also for other DAMPs released upon acute lung injury [22]. As such, our results argue against an important role for TLR4 as a DAMP receptor contributing to lung injury during *S. aureus* pneumonia. In contrast, earlier investigations reported a clear role for HMGB1-TLR4 signaling in sterile-injury models induced by ischemia-reperfusion [23] and trauma [24]. Remarkably, TLR4 seemed to protect against epithelial barrier disruption, as indicated by increased protein levels in BAL fluid of *tlr4*^*−/−*^ mice early after infection. Previous studies reported reduced epithelial barrier integrity in hyperoxia-induced lung injury in *tlr4*^*−/−*^ and C3H/HeJ mice by a mechanism that involved a diminished capacity to upregulate anti-apoptotic proteins [25,26]. Further studies are warranted to establish the mechanism by which TLR4 protects against alveolar protein leak during *S. aureus* pneumonia.

RAGE has been implicated in lung injury induced by hyperoxia [27] or bleomycin [28]. In addition, in pneumonia caused by *Streptococcus pneumoniae*, *rage*^*−/−*^ mice have shown mitigated lung injury and neutrophil migration [29]. Nonetheless, *Rage*^*−/−*^ mice showed only discreetly attenuated lung pathology at 6 hours after infection with *S. aureus* without alterations in neutrophil recruitment or protein leak. *Rage*^*−/−*^ mice did demonstrate reduced TNFα, IL-6 and KC concentrations in BAL fluid 24 hours post infection. Thus, although RAGE contributed to lung inflammation during experimental *S. aureus* pneumonia, its role was modest and was unlikely to be mediated by HMGB1. RAGE can interact with several other ligands including advanced glycation end-products, amyloid, β-sheet fibrils and members of the S100 protein family [9]. Which RAGE ligand(s) contribute to its effects in *S. aureus* pneumonia remains to be established. The current finding that *rage*^*−/−*^ mice displayed increased clearance of *S. aureus* from the bronchoalveolar compartment is in line with an earlier study from our laboratory reporting attenuated bacterial growth and dissemination in *rage*^*−/−*^ mice after induction of pneumonia by *Streptococcus pneumoniae*[29].

## Conclusions

In conclusion, we here describe distinct roles for HMGB1 and RAGE in lung injury accompanying the early phase of *S. aureus* pneumonia. HMGB1 is a proinflammtory mediator which is released in the bronchoalveolar compartment and contributes to protein leak and lung edema, while it does not influence neutrophil recruitment or bacterial clearance. Although TLR4 has been implicated as the dominant proinflammatory receptor for HMGB1 [10], this receptor had only limited impact on the injurious host response during *S. aureus* pneumonia. RAGE deficiency, however, was associated with reduced lung pathology and inflammation as well, suggesting that both HMGB1 and RAGE are harmful in the development of lung injury during the early phase of severe *S. aureus* pneumonia.

The current finding that neither anti-HMGB1 nor deficiency for the major DAMP receptors RAGE or TLR4 impact on the sustained lung pathology accompanying *S. aureus* pneumonia suggest that other DAMPs and receptors are involved. Candidates meriting further investigation include TLR3 (extracellular RNA), TLR9 (extracellular DNA) and the inflammasomes (multiple DAMPs).

## Key messages

• *S. aureus* pneumonia is associated with early (6 hours after infection) bronchoalveolar HMGB1 release, which contributes to inflammation, protein leak and lung edema

• TLR4 has only limited impact on the injurious host response during *S. aureus* pneumonia

• RAGE enhances pulmonary inflammation and pathology early after intranasal *S. aureus* infection

## Abbreviations

BAL: Bronchoalveolar lavage; CFU: Colony-forming units; CXCL1: C-X-C motif chemokine ligand 12; CXCR4: C-X-C chemokine receptor type 4; DAMPs: Damage-associated molecular patterns; ELISA: Enzyme-linked immunosorbent assay; H&E: Hematoxylin and eosin; HMGB1: High mobility group box 1; IgG2b: Immunoglobulin 2b; IL: Interleukin; KC: Keratinocyte-derived chemokine; MIP-2: Macrophage inflammatory protein; P. aeruginosa: *Pseudomonas aeruginosa*; PBS-T: Tween 20 phosphate-buffered saline; PRRs: Pattern recognition receptors; RAGE: Receptor for advanced glycation end products; Rage−/−: *Rage* deficient; S. aureus: *Staphylococcus aureus*; TLR: Toll-like receptor; Tlr4−/−: *Tlr4*-deficient; TNF-α: Tumor necrosis factor α; Wt: Wild-type.

## Competing interests

The authors declare that they have no competing interests.

## Authors’ contributions

AA participated in the design of the study, carried out the *in vivo* experiments, analyzed the data and drafted the manuscript; AJvdM carried out the *in vivo* experiments and helped draft the manuscript; SF performed pathology scoring, prepared part of the figures and helped draft the manuscript; HY and KJT provided critical reagents for *in vivo* experiments and helped draft the manuscript; CvV and AFdV participated in the design of the study, analyzed the data, advised in laboratory matters and helped draft the manuscript; TvdP participated in the design of the study, supervised the study and helped draft the manuscript. All authors read and approved the final manuscript.

## Supplementary Material

Additional file 1: Figure S1 *Receptor for advanced glycation end products (Rage) *^*−/−*^ show smaller areas of confluent inflammatory infiltrates at 48 hours after *S. aureus* infection. Representative slides of lung HE staining of wild-type (Wt) (**A**), *Toll-like receptor* (*tlr*)*4*^*−/−*^ (**B**) and *rage*^*−/−*^ mice (**C**) at 48 hours after infection, original magnification × 2. Scale bars indicate 200 μm. The percentage of the lung surface demonstrating confluent inflammatory infiltrate was determined in Wt (dark gray), *tlr4*^*−/−*^ (light gray) and *rage*^*−/−*^ mice (white) (**D**). Data are expressed as box-and-whisker diagrams depicting the smallest observation, lower quartile, median, upper quartile and largest observation (7 to 8 mice per group at each time point). *P <0.05 versus Wt mice at the same time point. Click here for file
