# Supplementary figures and images for: High-mobility group box 1 and the receptor for advanced glycation end products contribute to lung injury during Staphylococcus aureus pneumonia
Source: Crit Care. 2013 Dec 16;17(6):R296. doi: 10.1186/cc13162 (PMC4057161; doi:10.1186/cc13162)

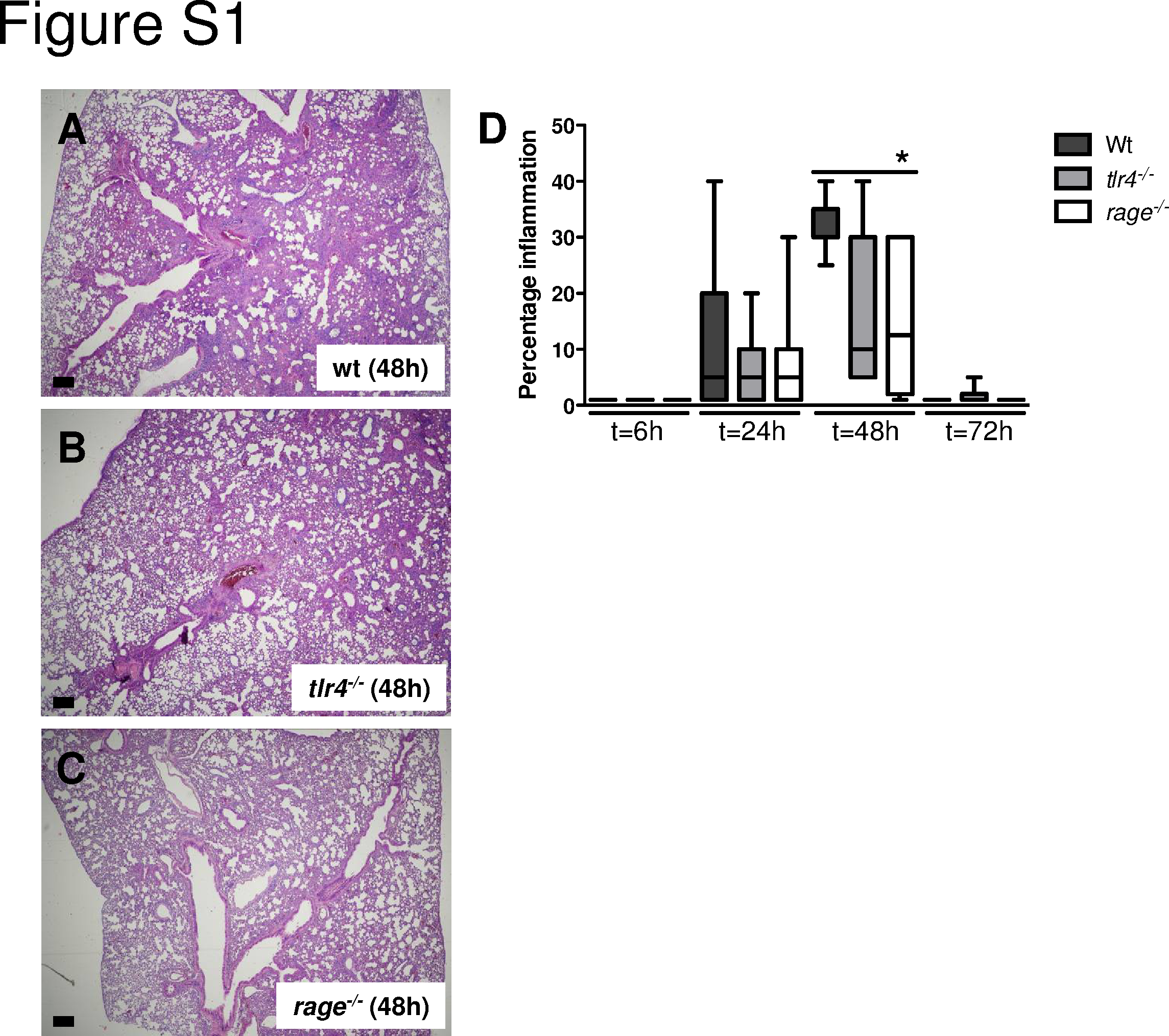

Supplement: Additional file 1: Figure S1 — Receptor for advanced glycation end products (Rage) −/− show smaller areas of confluent inflammatory infiltrates at 48 hours after S. aureus infection. Representative slides of lung HE staining of wild-type (Wt) (A), Toll-like receptor (tlr)4−/− (B) and rage−/− mice (C) at 48 hours after infection, original magnification × 2. Scale bars indicate 200 μm. The percentage of the lung surface demonstrating confluent inflammatory infiltrate was determined in Wt (dark gray), tlr4−/− (light gray) and rage−/− mice (white) (D). Data are expressed as box-and-whisker diagrams depicting the smallest observation, lower quartile, median, upper quartile and largest observation (7 to 8 mice per group at each time point). *P <0.05 versus Wt mice at the same time point. [file cc13162-S1.tiff]
